# Synthesis and preliminary *in vivo* evaluations of polyurethane microstructures for transdermal drug delivery

**DOI:** 10.1186/1752-153X-6-87

**Published:** 2012-08-14

**Authors:** Florin Borcan, Codruta M Soica, Srinivas Ganta, Mansoor M Amiji, Cristina A Dehelean, Melania F Munteanu

**Affiliations:** 1 Faculty of Pharmacy, “Victor Babes” University of Medicine and Pharmacy Timisoara, 2nd E. Murgu Sq., Timisoara, 300041, Romania; 2 Department of Pharmaceutical Sciences, Northeastern University, 360 Huntington Ave., Boston, Massachusetts, USA; 3 Department of Clinical Laboratory and Sanitary Chemistry, “Vasile Goldis” University, 1 Feleacului Str., Arad, 310396, Romania

**Keywords:** Polyurethane, Hollow microstructures, Lysine diisocyanate ester, Drug delivery, Zeta potential, CD1Nu/Nu mice, Noxiousness

## Abstract

**Background:**

Polymers have been considered as important materials in fabrication of microstructures for various medical purposes including drug delivery. This study evaluates polyurethane as material for hollow microstructures preparation.

**Results:**

Polyurethane microstructures were obtained by interfacial polyaddition combined with spontaneous emulsification and present slightly acid pH values. Scanning electron microscopy revealed the existence of irregular shapes and agglomerated microstructures. The material is heat resistant up to 280°C. Good results were recorded on murine skin tests in case of polyurethane microstructures based on isophorone diisocyanate. Mesenchymal stem cells viability presents good results for the same sample after 48 hours based on the Alamar Blue test.

**Conclusions:**

The research revealed the reduced noxiousness of this type of microstructures and consequently the possibility of their use for therapeutic purposes.

## Background

Many types of microstructures are used as carriers for therapeutic delivery of active substances. In this field, molecules such as chemotherapeutic agents can be selectively attached to the microstructure surface or interior, by covalent conjugation or encapsulation. Another possibility consists in polymer coatings of the drug in order to improve its physical-chemical or biological properties [1,2]. The major classes of nano- and/or micro-structures used for biomedical applications include: liposomes, nanoshells, metals and metal oxides, carbon-based particles, nano- and/or micro-emulsions, crystals and polymer-based nano- and/or micro-materials [2,3]. Polymeric nano- and/or micro-structures can be prepared by dispersion of pre-formed polymers or by polymerization [4], the second being preferred because it implies fewer purifying procedures to eliminate organic solvents. Polymerization methods implicated in nano- and/or micro-structure preparation depend on the type of the monomer and polymerization mechanism, emulsion polymerization being probably the most frequently used [5].

Polyurethanes (PU) consist in hard segments (which alternate diisocyanate and chain-extender molecules) and amorphous soft, linear diol segments [6]. Within the hard tissue engineering, much emphasis has been put on developing lysine-diisocyanate (LDI) based polyurethanes into load-bearing scaffolds [7]. Bonzani *et al.* developed a novel two-component injectable scaffold system [8] which presented better properties than most other injectable systems, thus improving osteoblast viability and proliferation.

The use in the therapeutical field imposes the necessity of toxicological evaluation of any new delivery system. One US Patent of H. Ralph Snodgrass describes a method for toxicity evaluation based on mesenchymal stem cells (MSCs); in his study mammalian MSCs were used and molecular profiles were correlated with the chemical compositions’ noxiousness [9].

It is well-known that polyurethane materials based on aromatic diisocyanates suffer an *in vivo* degradation leading to carcinogenic aromatic amines [10]. The present study avoids this issue by developing three types of PU microstructures which are based on aliphatic diisocyanates and designed for transdermal drug delivery. The aim of this research was to obtain microstructures with a diameter of 600 nm and to test their possible biological application using preliminary *in vitro* and *in vivo* tests. We studied the influence of raw material structure on microstructures’ size and we tested their possible noxiousness on MSCs and also mouse skin which is more permeable for penetration of topic compounds. Hairless mice can be used in such evaluations offering a convenient and reliable model because of their sensitive skin [11].

## Results and discussion

The pH values of microstructures solutions were measured with a Schott TitroLine by simply plunging the electrode into the PU aqueous solutions (1:5000 v/v). The samples present slightly acid pH values (6.61 for sample PU_1, 6.13 for sample PU_2, and 6.44 for sample PU_3) due to the characteristics of its components. The absence of secondary products (amines) is demonstrated by the weak acid character of suspensions. Beside, these pH values are appropriate for products intended for cutaneous administration [12].

The synthesis of different hollow polymer microstructures for drug delivery field is nowadays a research topic of high interest [13,14]. The aim of this study was the synthesis and testing of PU microstructures with low toxicity and controlled-sized particles. For drug delivery systems small size particles are very important in order to overcome the defence barrier of corneous layer [15,16], but on the other hand the quantity of encapsulated drug must be taken into consideration. In the PU microstructures synthesis the presence of an initiator to form the chains is not necessary. In this case the assembly of polymer chains and the formation of the polymer shell take place simultaneously, thus it is called an *in-situ* polymerization method [17]. In principle, the method can be adopted in systems where the monomer is soluble in water while the corresponding polymer is insoluble in water at the reaction conditions.

**Figure 1:**
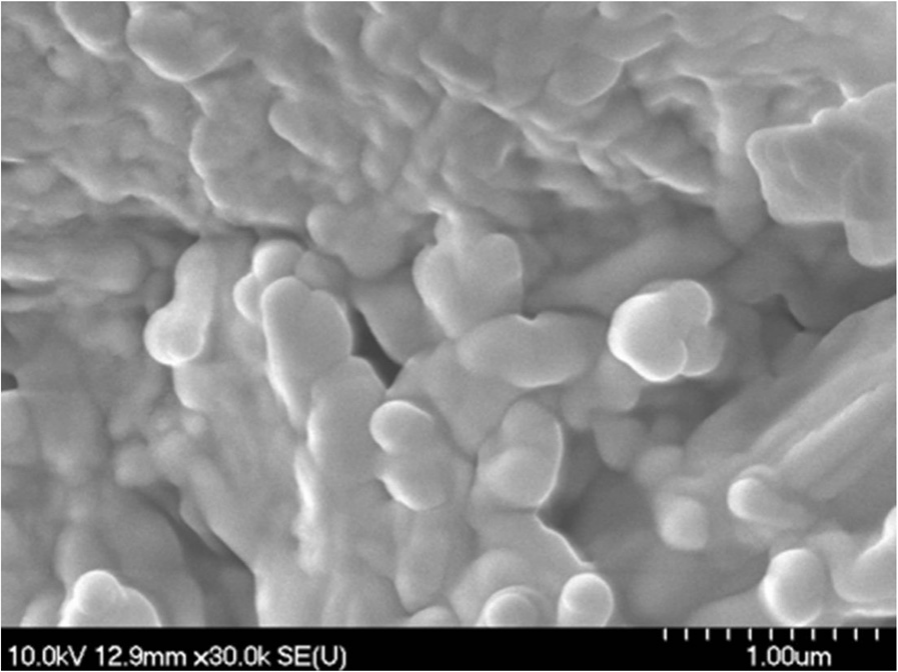
SEM images of PU1 microstructures.

**Figure 2:**
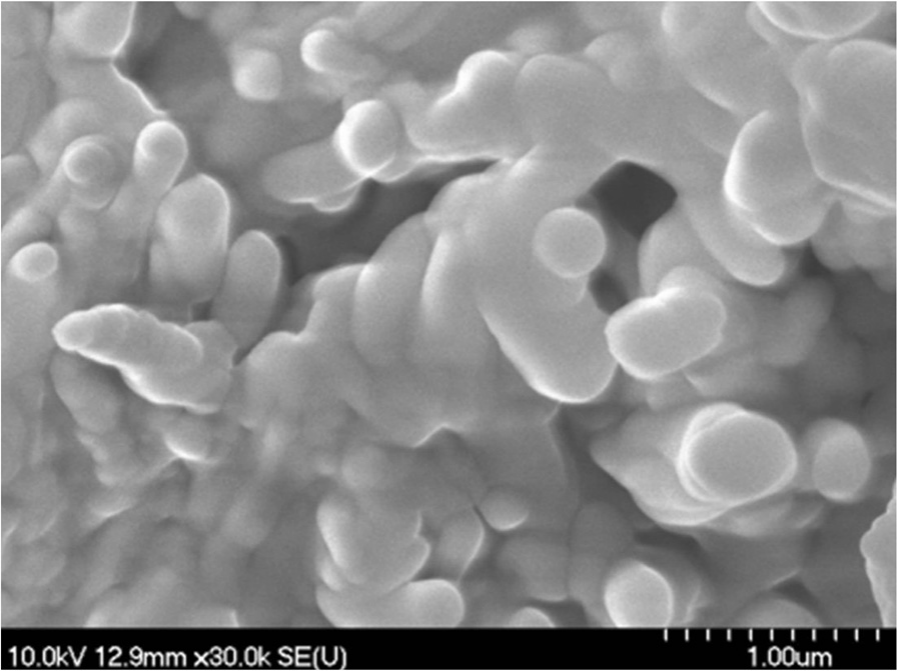
SEM images of PU_2 microstructures.

**Figure 3:**
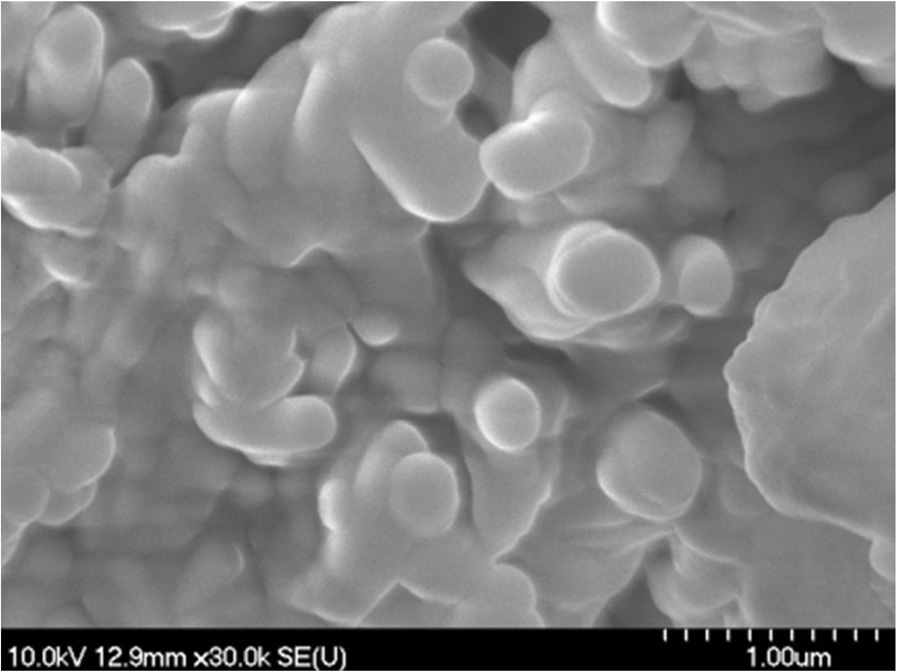
SEM images of PU_3 microstructures.

During scanning electron microscopy (SEM) investigations, the existence of microstructure aggregates of irregular shapes was detected (Figures 1, 2 and 3); the shape and the size are not influenced by the diisocyanate used. The microstructures are not spherical in shape which consequently leads to a poor flowing ability. The morphology of these PU agglomerates is not characteristic for a porous-type material which represents an advantage for an intended transdermal vehicle in order to protect its load. Non-agglomerated microstructures cannot be successfully synthesized probably due to their low mass.

**Figure 4:**
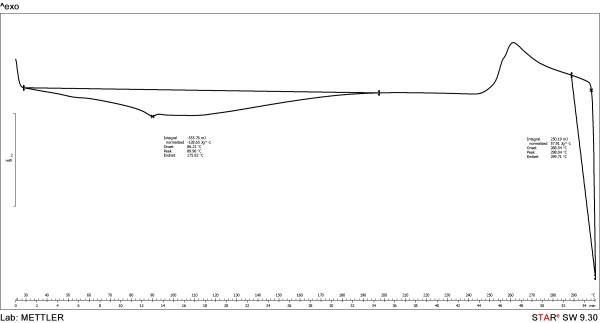
The thermal decomposition behaviour of PU_1 microstructures.

**Figure 5:**
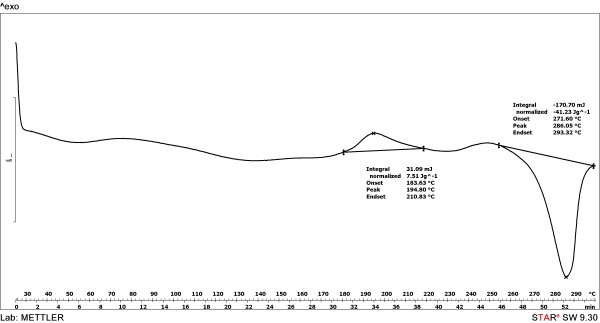
The thermal decomposition behaviour of PU_2 microstructures.

**Figure 6:**
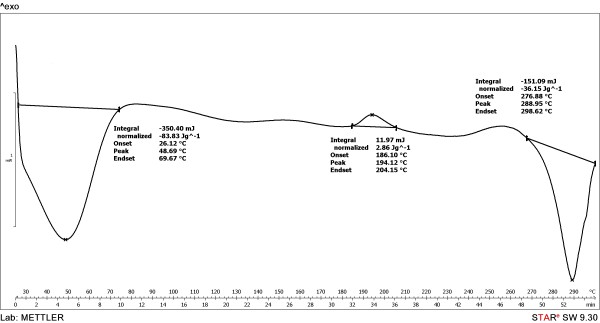
The thermal decomposition behaviour of PU_3 microstructures.

Differential scanning calorimetry (DSC) showed that the degradation is due to a thermooxidative process (exothermic effect) and takes place at temperatures significant higher than the melting point of the polymer (Figures 4, 5 and 6). As observed, the initial temperatures of the thermooxidative process were closed, as follows: PU_1 (297°C) > PU_3 (281°C) > PU_2 (276°C). The final temperatures were: 292 and 296°C for PU_2 and PU_3, respectively. Also, one can notice the absence of glass transition temperature which is determined for most polymers; its measurement depends on a crystalline transition so its absence usually reveals the predominant amorphous nature of a polymer [18].

The Zetasizer results (Table 1) show large values for the diameter of each sample (over 500 nm) which could be attributed to the particle aggregation process. The zeta potential values are significant because if all the particles have a zeta potential more negative than −30 mV or more positive than +30 mV the dispersion should remain stable [19]. All this considered, the product obtained in the second experiment, when isophorone diisocyanate (IPDI) was used, is regarded as the most stable.

**Table 1:** The Zetasizer characterization for the PU microstructures

**Sample code**	**Particle size (nm)**		**Zeta Potential (mV) Mean ± SD**
	**Mean ± SD**	**Polydispersity index**	
PU_1	601 ± 142	0.5	18.4 ± 6.2
PU_2	683 ± 242	0.3	27.5 ± 0.4
PU_3	643 ± 201	0.6	13 ± 1.0

Noxiousness investigations were carried out on mesenchymal stem cells; sources of stem cells described in the literature are: bone marrow, peripheric blood, citaferesis concentrate, umbilical cord and placenta [20]. The main source for MSCs and haematopoetic cells remains bone marrow which was also used in our experiments.

All experiments were done in quadruplicate so the absorbance average of four wells was calculated. Values thus obtained were introduced into the following formula in order to calculate the reduction level of Alamar Blue reagent by metabolic activity of cells:

$$AB\%N=\left[\left(O2\;A1-O1\;A2\right)/\left(R1\;N2-R2\;N1\right)\right]\;100$$

where AB%N is the percentage of reduction of Alamar Blue reagent compared to the negative control (medium without cells); O1 represent molar extinction coefficient (E) for oxidized Alamar Blue at 570 nm; O2 is molar extinction coefficient (E) for oxidized Alamar Blue at 600 nm; R1 is molar extinction coefficient (E) for reduced Alamar Blue (red) to 570 nm; R2 is molar extinction coefficient (E) for reduced Alamar Blue to 600 nm; A1 represent absorbance of tested cells at 570 nm; A2 is absorbance for tested cells at 600 nm; N1 is absorbance of negative control (medium with Alamar Blue and without cells) to 570 nm; N2 is absorbance of negative control (medium with Alamar Blue and without cells) to 600 nm [21]. The results are expressed as the mean for each quadruplicate culture ± the standard error.

A high percentage of reduced Alamar Blue (red) reactive indicates a strong metabolic activity and consequently an increased viability and cell proliferation, while unviable cells produce an oxidized medium (blue). Previous studies made on silicon-based microparticles have shown that surface properties and chemistry may influence the adhesion and function of cells in culture [22].

**Figure 7:**
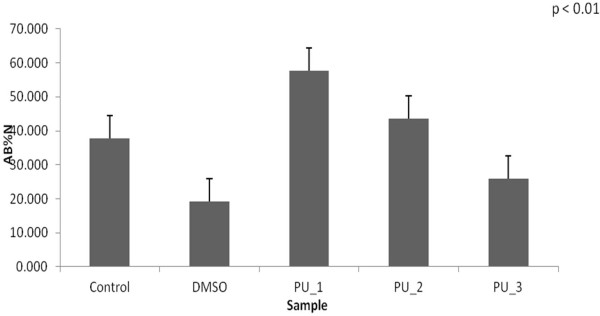
The MSCs viability after 24 hours.

**Figure 8:**
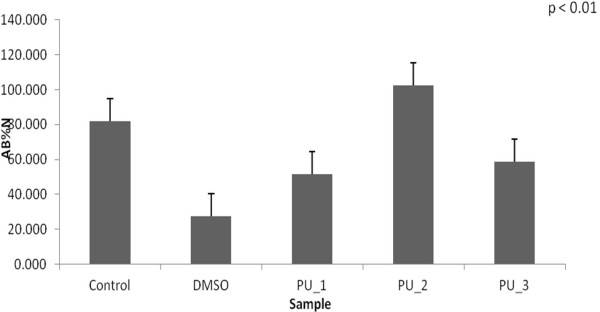
The MSCs viability after 48 hours.

All samples exhibited metabolic activity as measured by the detected fluorescence of the Alamar Blue reagent after 2 hours incubation at 37°C. Alamar Blue results were significantly different between day 1 and 2 (Figures 7 and 8), but cells were still viable throughout the experiment.

A good result for MSCs viability was recorded for PU_1 after one day, but we consider MSCs viability after 48 hours to be more important. In this case, the best value was recorded in case of PU microstructures based on IPDI. The main conclusion Alamar Blue test produced was the reduced noxiousness for the studied polymeric microstructures.

CD1Nu/Nu (nude mice) present an abnormal hair growth, due to functional follicles but failed hair growth and that is why the skin gives a quick response to any external aggressive factor. This mice type was chosen for our experiment because their skin has the following important features: it is very sensitive and exhibits a penetration degree a few times higher than human skin [23,24]. The sensitivity of these mice’ skin is an advantage as it can be used as a parameter characterizing the investigational product noxiousness. In this study, Tween®20 and diisocyanates by their specific group (−N = C = O) are known as dangerous substances for human health [25,26], but on the other hand we have demonstrated above that the polyurethane products did not reveal any toxicity. In the present work an excess of polyol was used in order to avoid the diisocyanates toxicity issue, excess easily removed by washing and useful to prevent formation of secondary amine products.

The transepidermal water loss (TEWL) for the hairless mice was evaluated by non-invasive techniques for the characterization of skin changes [27].

**Figure 9:**
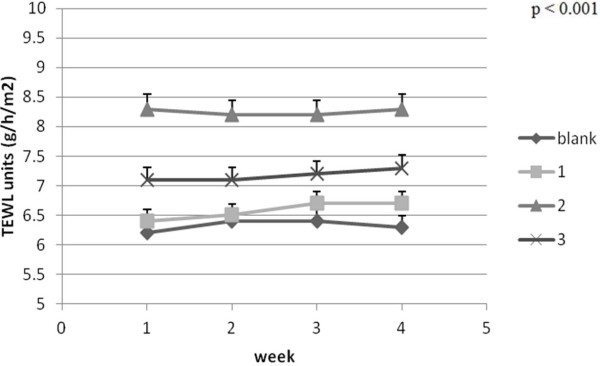
TEWL evolution ± SEM: TEWL, pH, melanin, and erythema.

Because the application of a new topical compound could determine important skin parameter changes (e.g. erythema), the assessment of local haemoglobin and secondary melanin (for pigmented animals) content could be helpful. The Courage-Khazaka instruments are manufactured to be generally used in cosmetic tests on human skin. This is the reason why, previous to this research, we created a database (unpublished) with mouse skin parameter values (measured on batches consisting of four different species of mice, of various ages, with and without skin lesions). In this study, the evolution of TEWL values for the four CD1Nu/Nu mice batches (one blank group and three groups corresponding to the applied PU microstructures) lies in the range 0–10 g/h/m^2^ which is specific to skin in very good condition (Figure 9) [28]. The mice batch where PU_2 was applied shows a stationary trend over the four weeks so one can conclude that IPDI based PU microstructures are appropriate ingredients for skin application. In other cases TEWL values slightly increase indicating a reduced damage. Increased TEWL values indicates a skin dehydration process [29] but if the values are not very high above the limit increased TEWL values do not indicate a certain sign of noxiousness.

**Figure 10:**
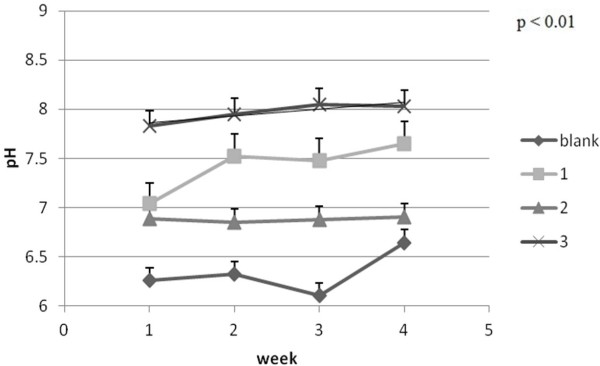
Skin-pH evolution ± SEM.

The measured pH average values for CD1Nu/Nu mice skin are between 6 and 8. One can notice in Figure 10 that batches 2 and 3 (where PU_2 and PU_3 were applied) least change their values during the experiment. Measured pH values indicated no important changes at skin level [30] leading to the conclusion that application of these PU formulations maintained the pH of skin.

**Figure 11:**
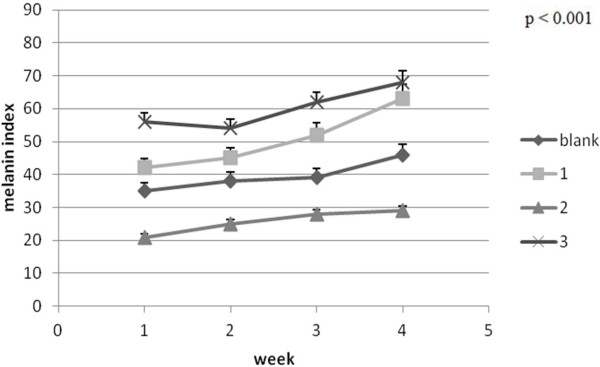
Melanin evolution ± SEM.

**Figure 12:**
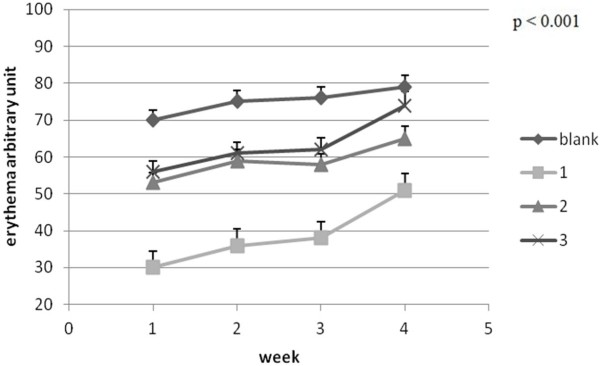
Erythema evolution ± SEM.

The erythema, measured by the Mexameter®MX 18 probe, is a parameter which slightly increases its values during different skin testing [31]. When erythema values change rapidly and significantly it usually is an indicator of an important skin stress such as skin injury, infection or inflammation [32]. In this experiment the lowest change of the erythema (local haemoglobin) values was in case of mice batch 2 (where PU_2 was applied) which recommends this sample as a transdermal carrier with the most reduced noxiousness Figures 11 and 12.

## Conclusions

The interfacial polyaddition technique combined with spontaneous emulsification is an adequate procedure for PU microstructures synthesis. The microstructures suspensions present appropriate pH values for products intended for cutaneous administration. The use of three different diisocyanates (HMDI, IPDI and LDI) in the synthesis of PU microstructures was proved as a way to obtain microstructured polymers which can successfully candidate as transdermal drug delivery systems. The comparative analysis of these three diisocyanates performance revealed that the IPDI based microstructures are the most stable formulations which also present the best toxicological profile as revealed by MSCs and *in vivo* tests.

## Methods

### Synthesis procedure

Lysine diisocyanate ester (LDI) was obtained from Hangzhou Imaginechem Co., Ltd (China). Mono-ethylene glycol (MEG) was purchased from Lach-Ner s.r.o. (Czech Rep.) while 1,4-butanediol (1,4-BD) was purchased from Carl Roth GmbH (Germany). All the other raw materials, hexamethylene diisocyanate (HMDI), isophorone diisocyanate (IPDI), polyethylene glycol (PEG 200), the solvent (acetone) and surfactant (Tween®20) were obtained from Merck (Germany). All substances were used as received.

The polyaddition between diols and diisocyanates for the polyurethane synthesis follows the reaction from Scheme 1. Hydroxylic and isocyanate components were used in a ratio of 1.1 : 1 (w/w) because the hydroxyl excess is easily removed by washing. However, the product yield from secondary reactions (amines) was reduced.

The procedure for obtaining PU microstructures by interfacial polyaddition combined with spontaneous emulsification is based on K. Bouchemal experiment [33] and involves the following steps:

1. Preparation of the organic solution - 1.6 mL diisocyanate is mixed with 20 mL acetone in a Berzelius beaker (50 mL) and heated at 40°C.

2. Preparation of the homogeneous aqueous phase - 0.6 mL MEG, 0.6 mL 1,4-BD, 1.2 mL PEG 200 and 1.5 mL Tween®20 are mixed with 40 mL distilled water in an Erlenmeyer flask (100 mL) and heated at 40°C.

3. Organic phase is injected into the aqueous phase at 40°C under constant magnetic stirring (500 rpm). Microstructures are formed during this step.

4. Stirring is continued for four hours at 40°C in order to ensure the maturation of the microstructure walls.

5. Solvent (acetone) as well as a part of water is removed by slow evaporation in the oven, keeping the suspension as thin layers (approx. 3 mm) in Petri dishes at 60°C for 12 hours.

6. Resulting products are purified by three cycles of centrifugation and dispersion in a mixture water-acetone (1:1, v/v) in order to eliminate possible secondary products (amines).

Three experiments were done using the same procedure already described. Three diisocyanate with different aliphatic chains were chosen to study the effect over the microstructures size and stability (Table 2).

**Table 2:** The raw materials ratio for PU microstructures synthesis

**Sample code**	**Diisocyanate, mL**	**Aqueous phase**			
		**MEG, mL**	**1,4-BD, mL**	**PEG, mL**	**Tween® 20, mL**
PU_1	HMDI, 1.6	0.6	0.6	1.2	1.5
PU_2	IPDI, 1.6	0.6	0.6	1.2	1.5
PU_3	LDI, 1.6	0.6	0.6	1.2	1.5

After the samples were well dried, the solubility and pH of microstructures were measured at the same concentration.

### Scanning electron microscopy (SEM)

PU microstructures’ shape and morphology were examined using a scanning electron microscope Hitachi 2400S (Hitachi Scientific Ltd., Japan) using a voltage of 10 kV. A thin-layer covering device (Bio-Rad SC 502, VG Microtech, England) was used to obtain an electric conductivity to the surface of the samples. The air pressure was between 1.3-13.0 mPa.

### Differential scanning calorimetry

Thermal analysis was carried out with a Mettler-Toledo 821e instrument between 30–300°C because the urethane group (−NH-COO-) is stable in this temperature range [34].

### Size and surface charge measurements

Particle size, polydisperisity index and zeta potential of the microstructures were measured using a Zetasizer Nano ZS (Malvern, UK). Microstructures suspended in deionized distilled water were added to the sample cuvette and the particle size was measured at a 90° fixed angle and at 25°C. Zeta potential (surface charge) of the suspended microstructures was also measured using a Zetasizer Nano ZS. For this, microstructures suspension was diluted in deionized distilled water and placed in the electrophoretic cell, and the measurements were done at 25°C under electric field strength of 23.2 V/cm. The measurements were carried out three times for each sample.

### Mesenchymal stem cells (MSCs) viability tests

Bone marrow was prelevated from patients admitted to Orthopedics, County Clinical Hospital no. 1 Timisoara, Romania, who have been submitted for bone surgery. Previously to prelevation, patients have been informed about the future use of the biological sample and they all agreed and signed an Informed Consent according to the Helsinki Declaration of WHO. The prelevation was carried out under sterile conditions, using heparinated syringes.

Approximately 10 mL bone marrow, as source for MSCs, was diluted 1:1 with phosphate buffer (PBS, Sigma), centrifuged (1500 rpm) and placed with proliferation medium consisting of MEM Alpha medium (MEM – Alpha; Gibco BRL, Invitrogen, Carlsbad, CA, USA) supplemented with 10% fetal calf serum, (FCS, PromoCell, Heidelberg, Germany), 10 ng/mL fibroblast growth factor (FGF, Sigma, St. Louis, USA) and 2% mixture of penicillin / streptomycin (10 000 IU/mL, PromoCell, Germany) in plastic culture plate specific for adherent cell culture (Falcon, 75 cm^2^, Becton Dickinson). Culture plates were incubated at 37°C; medium was replaced after 48 hours with fresh medium and the first germ centers with the first colonies of plastic adherent fibroblast cells could be seen using a reversed microscope. Plates were washed after 7 days using PBS (Dulbecco’s Phosphate Buffered saline, Sigma), followed by a slight rinsing and the confluence cell level was observed. Medium was replaced every 3–4 days and the cells were passed when reached 80-90% confluence.

The culture plate was washed with PBS to remove traces of culture medium and preheated Trypsin-EDTA 0,25% (Sigma) was added in order to act for 2 minutes on incubated cells after which the microscope was used to observe the separation of cells. After MSCs were removed from the plate, trypsin and cells suspension together with trypsin inactivation medium were centrifuged; the supernatant was removed and cells were counted with a hemocytometer, using 0,4% Trypan Blue (Sigma), as vital dye. Depending on the number of cells, they were distributed and reinsaminated in other culture plates at 10,000 cells/cm^2^ to ensure optimal proliferation. MSCs obtained were passed, expanded and further used in experiments only starting with passages 4–5, at each passage some cells being frozen for future use. Long period storage of cells is being done in liquid nitrogen, in special tanks.

CellTiter-Blue method was used to estimate the viability of cells present in wells plates. It uses resazurin as color dye to measure the metabolic capacity of cells - an indicator of cell viability. Viable cells have the ability to reduce resazurin to resorufin, a fluorescent product. Unviable cells rapidly lose metabolic capacity, do not reduce the dye, and therefore do not generate a fluorescent signal.

After the cells were counted and trypsinized, it were seeded in 96 wells plate at a concentration of 7000 cells / well in 150 μL culture medium / well. Thereafter the cells were allowed to join the incubator at 37°C and 5% CO_2_ in their proliferation medium for approximately 24 hours. Next day, cell medium was replaced with medium supplemented with transdermal carrier based on polyurethane microstructures in concentration 0.1 mg / ml. All samples were placed in quadruplicate and also were made four wells with medium with DMSO (1.5 μl) as negative control and control wells (blank wells or standard) with normal medium for cell proliferation and wells with only medium without cells. After 24 and 48 hours of cells exposure to the mixture of medium with trandermal carrier, their viability was tested by CellTiter-Blue method. It was added to each well of 96 wells plate, a volume of 15 μl Alamar Blue solution, which represent 10% of the medium volume in each well. Plate was further incubated at 37°C for 4–10 hours until there was observed a color reaction, changing of Alamar Blue reagent from blue color (resazurin) to pink (resorufin). After incubation the absorbance was measured at 570 nm and 600 nm wavelengths, using a spectrophotometric plate reader (Bio-Rad, Tokyo, Japan).

### Animals

CD1Nu/Nu mice of eight weeks were purchased from Charles River (Sulzfeld, Germany). The work protocol followed all NIAH - National Institute of Animal Health rules. Animals were maintained during the experiment in standard conditions of 12 hours light–dark cycle, food and water *ad libitum*, temperature 24°C, humidity above 55%. Mice were divided in four groups (one blank and one group for each PU microstructure type).

### Measurements of skin parameters

In order to observe the changes of the skin parameters, PU microstructures suspensions (2%, w/w) were applied on the back skin of CD1Nu/Nu mice for four weeks, three times a week (every other day) (50 μL solution / application).

After each application, skin parameters determination was performed within 30 minutes. All the measurements on the mice skin were carried out according to the published guidelines [35] with a Multiprobe Adapter System (MPA5) from Courage&Khazaka Electronics, Germany, equipped with a Tewameter®TM300 probe, a Skin-pH-meter®PH905 probe, and a Mexameter®MX18 probe.

Measurements with the Skin-pH-meter®PH905 are made using a probe consisting of a single glass rod containing all the sensor elements. The planar design of the probe head allows direct measurement on the skin. The short measuring time avoids occlusive effects on the skin [36].

The melanin values are between 0 and 999 and the measurement is based on the absorption/reflexion. The protocol followed the observations on haemoglobin status (pigmentation and erythema). The device was applied on most obvious affected areas and maintained on the skin for 10 seconds [37].

Measurements of TEWL or TEWA (transepidermal water loss) is an useful tool for revealing and evaluating skin damage of chemical, physical or pathological origin as there is a direct proportionality between TEWL increase and the level of damage. This probe records values between 0–10 g/h/m2 for skin in very good condition, 10–15 g/h/m2 skin in good condition, 15–25 g/h/m2 for normal skin, 25–30 g/h/m2 for skin in bad condition and over 30 g/h/m2 for very bad conditions [38].

The data were recorded by the specific soft from the Mexameter®MX18 probe and then expressed as arbitrary units. All data were processed as initial and final measurements values on the same area.

## Competing interests

The author(s) declare that they have no competing interests.

## Authors’ contributions

FB and CMS carried out the synthesis, and purification of PU microstructures. SG and MMA carried out the Zetasizer measurements. CAD carried out the MSCs and mouse skin tests, conceived the study, and participated in its design and coordination and helped to draft the manuscript. MFM have helped to review the paper. All authors read and approved the final manuscript.
